# The benefits and harms of providing parents with weight feedback as part of the national child measurement programme: a prospective cohort study

**DOI:** 10.1186/1471-2458-14-549

**Published:** 2014-06-03

**Authors:** Catherine L Falconer, Min Hae Park, Helen Croker, Áine Skow, James Black, Sonia Saxena, Anthony S Kessel, Saffron Karlsen, Stephen Morris, Russell M Viner, Sanjay Kinra

**Affiliations:** 1 Bristol Biomedical Research Unit in Nutrition, Diet and Lifestyle, Level 3, University Hospitals Bristol Education Centre, Bristol BS2 8AE, UK; 2 Department of Non-communicable Disease Epidemiology, London School of Hygiene and Tropical Medicine, Keppel Street, London WC1E 7HT, UK; 3 Health Behaviour Research Centre, UCL Department of Epidemiology and Public Health, University College London, 1-19 Torrington Place, London WC1E 6BT, UK; 4 Department of Primary care and Public Health, Imperial College London, Reynolds Building, St Dunstan’s Road, London W6 8RP, UK; 5 Faculty of Public Health and Policy, 15-17 Tavistock Place, London School of Hygiene and Tropical Medicine, London WC1H 9SH, UK; 6 UCL Department of Applied Health Research, University College London, 1-19 Torrington Place, London WC1E 7HB, UK; 7 UCL Institute of Child Health, University College London, 30 Guilford Street, London WC1N 1EH, UK

**Keywords:** Childhood obesity, Weight feedback, National child measurement programme, BMI screening

## Abstract

**Background:**

Small-scale evaluations suggest that the provision of feedback to parents about their child’s weight status may improve recognition of overweight, but the effects on lifestyle behaviour are unclear and there are concerns that informing parents that their child is overweight may have harmful effects. The aims of this study were to describe the benefits and harms of providing weight feedback to parents as part of a national school-based weight-screening programme in England.

**Methods:**

We conducted a pre-post survey of 1,844 parents of children aged 4–5 and 10–11 years who received weight feedback as part of the 2010–2011 National Child Measurement Programme. Questionnaires assessed general knowledge about the health risks associated with child overweight, parental recognition of overweight and the associated health risks in their child, child lifestyle behaviour, child self-esteem and weight-related teasing, parental experience of the feedback, and parental help-seeking behaviour. Differences in the pre-post proportions of parents reporting each outcome were assessed using a McNemar’s test.

**Results:**

General knowledge about child overweight as a health issue was high at baseline and increased further after weight feedback. After feedback, the proportion of parents that correctly recognised their child was overweight increased from 21.9% to 37.7%, and more than a third of parents of overweight children sought further information regarding their child’s weight. However, parent-reported changes in lifestyle behaviours among children were minimal, and limited to increases in physical activity in the obese children only. There was some suggestion that weight feedback had a greater impact upon changing parental recognition of the health risks associated with child overweight in non-white ethnic groups.

**Conclusions:**

In this population-based sample of parents of children participating in the National Child Measurement Programme, provision of weight feedback increased recognition of child overweight and encouraged some parents to seek help, without causing obvious unfavourable effects. The impact of weight feedback on behaviour change was limited; suggesting that further work is needed to identify ways to more effectively communicate health information to parents and to identify what information and support may encourage parents in making and maintaining lifestyle changes for their child.

## Background

The National Child Measurement Programme (NCMP) is a school-based weight surveillance initiative established in 2006 by the Department of Health for England as part of the UK Government’s ‘Healthy Weight, Healthy Lives’ strategy [1]. The NCMP monitors the heights and weights of children in state primary schools each year, at school entry (Reception, ages 4–5 years) and in Year 6 (ages 10–11 years), and provides written feedback to parents about their child’s weight status. The specific aims of the NCMP are to provide local surveillance data to set and monitor goals for tackling child obesity, and for local services to engage with parents and carers through the provision of weight feedback.

Few parents of overweight and obese children recognise that their child is overweight [2,3]. Providing parents with accurate information about their child’s weight status may improve perceptions or recognition of overweight, and encourage parents to make positive lifestyle changes for their children [4,5]. However, there are concerns that identifying a child as overweight could lead to weight-related teasing and parental distress [6].

There is limited evidence that providing written weight feedback to parents promotes behaviour change. A small-scale study from the United States (US) showed that written feedback can improve parental awareness of their child’s overweight status [7]. Evaluation of a pilot written weight feedback programme in England indicated that feedback was not associated with increased recognition of child overweight, but some parents reported positive changes in diet and physical activity [5]. An early assessment of written NCMP feedback showed that a third of parents planned to make lifestyle changes as a result of the feedback [8]. However, this small-scale cross-sectional study did not assess actual behaviour change, nor did it examine which groups may benefit the most from feedback. BMI screening in school is a controversial approach and has received much media attention in the UK. In 2007, a review concluded there to be a lack of evidence for the effectiveness of BMI screening and without effective weight reduction initiatives for children in the UK, BMI screening for obesity was considered difficult to justify [9].

Despite the lack of prospective evidence on the effects of this approach, over one million children were measured as part of the NCMP in 2010–2011, and their parents were provided with feedback. Our aims were to assess the effects of NCMP feedback on parents and children, and to identify whether these effects vary by the characteristics of the participants or type of feedback.

## Methods

### Study characteristics

We established a cohort of parents of children enrolled in the NCMP in five Primary Care Trusts (PCTs) in England who were undertaking the NCMP between May-July 2010–2011 [10]. PCTs are administrative bodies responsible for the commissioning and delivery of primary care services to local areas in the UK. In early 2013, PCTs were dissolved and the NCMP is now under the responsibility of Local Authorities (LA). PCTs were purposively selected to provide a representative sample of children participating in the NCMP in terms of ethnicity, deprivation and prevalence of overweight and obesity.

Questionnaires were administered at baseline (before feedback), and at one month and six months after weight feedback. Parents of all children participating in the NCMP between the months of May and July 2010–2011 in Redbridge, Islington, West Essex, Bath and North East Somerset (BANES) and Sandwell PCT (n = 18,000) were invited to participate in the study. Self-administered baseline questionnaires were distributed through schools on the day of the measurement, from February to July 2011. Follow-up questionnaires were mailed to all parents who completed a baseline questionnaire. Ethical approval was obtained from the London School of Hygiene and Tropical Medicine Ethics Committee.

### NCMP measurement and feedback

PCTs carried out their usual measurement and feedback procedures. In brief, PCTs sent letters to parents outlining the aims of the NCMP and the measurement process, and provided an opportunity for parents to withdraw their child from the measurement. Eligible children had their heights and weights measured at school by trained staff. Within six weeks of the measurement, written feedback was mailed to parents with information about their child’s body mass index (BMI) category, defined using centiles of the United Kingdom (UK) 1990 growth curves; clinical cut-offs at the 2^nd^, 91^st^ and 98^th^ BMI centiles defined underweight, healthy weight, overweight and obese (described to parents as ‘very overweight’), respectively (Table 1) [11]. Parents of overweight and obese children were provided with information about the health risks associated with their child’s weight status. Feedback also included information about healthy lifestyles from the Department of Health’s Change4Life campaign [12], and information about local health and leisure services. Redbridge, BANES and Sandwell PCTs supplemented the written feedback with school nurse-led telephone calls to parents of children identified as obese (‘proactive feedback’), in which parents were able to discuss the written feedback and seek advice. Parents in Redbridge PCT were also offered a face-to-face appointment with a school nurse.

**Table 1:** Wording from specimen result letters to parents and carers by child’s weight category (NCMP operational guidance 2010/2011)

**Weight category**	**Summary paragraph**
Underweight	Your child’s result is in the underweight range.
	We wanted to let you know your child’s result because it is an important way of checking how your child is growing.
	Many underweight children are perfectly healthy, but sometimes it can mean they have a health problem.
	Some parents find it help to re-check their child’s BMI after a few months, to see if they have moved into the healthy range as they grow. You can do this using the Healthy Weight tool at: http://www.nhs.uk/Tools/Pages/Healthyweightcalculator.aspx
	If you would like to speak to us about your child’s result, please phone the number at the top of this letter.
Healthy weight	Your child’s result is in the healthy range.
	We wanted to let you know your child’s result because it is an important way of checking how your child is growing.
	Children of a healthy weight are more likely to grow into healthy adults. To keep growing healthily, it is important that your child eats well and is active.
	Some parents find it helpful to re-check their child’s BMI after a few months, to see if they remain in the healthy range as they grow. You can do this using the Healthy Weight tool at: http://www.nhs.uk/tools/pages/healthyweightcalculator.aspx
	Many parents have found the tips in the enclosed leaflet and at http://www.nhs.uk/change4life useful in helping them make changes to help their child grow healthily. If you would like more advice about your child’s eating or activity, visit http://www.nhs.uk/change4life or phone the number at the top of this letter.
Overweight	You may be surprised that your child’s result is in the overweight range.
	It can sometimes be difficult to tell if your child is overweight as they may look similar to other children of their age, but more children are overweight than ever before.
	Research shows that if your child is overweight now, they are more likely to grow up to be overweight as an adult. This can lead to health problems. So this measurement is an important was of checking how your child is growing.
	Many parents have found the tips in the enclosed leaflet and at http://www.nhs.uk/change4life useful in helping them make small lifestyle changes to keep their child in the healthy weight range.
	Some parents also find it helpful to re-check their child’s BMI after a few months, to see if they remain in the healthy range as they grow. You can do this using the Healthy Weight tool at: http://www.nhs.uk/tools/pages/healthyweightcalculator.aspx
	If you are concerned about the result and would like further information and to find out about local activities, please phone us on the number at the top of this letter.
Very overweight	Your child’s result is in the very overweight range. Doctors call this clinically obese. We wanted to let you know your child’s result because it is an important way of checking how your child is growing.
	Children who are very overweight are more likely to have health problems at a young age, such as high blood pressure, early signs of type 2 diabetes and low self-confidence. Later in life, they are more likely to have illnesses like heart disease and some types of cancer.
	Small lifestyle changes started now can help your child to grow healthily. Many parents have found the tips in the enclosed leaflet and at http://www.nhs.uk/change4life useful in helping them make changes to help their child grow healthily.
	Some parents also find it helpful to re-check their child’s BMI after a few months, to see if they have moved towards the healthy range as they grow. You can do this using the Healthy Weight tool at: http://www.nhs.uk/tools/pages/healthyweightcalculator.aspx
	If you are concerned about the result and would like further information, please phone us on the number at the top of this letter.

### Main outcome measures

The following outcomes were assessed before and after NCMP feedback: 1) Parental knowledge of childhood obesity as a health problem, assessed using the question, ‘Do you think that being overweight increases a child’s future risk of any of the following: diabetes, cancers, heart disease, high blood pressure, and arthritis?’ Parents that correctly identified four or more conditions were considered to have good knowledge; 2) Child’s diet, based on parent-reported frequency of consumption of fruits, vegetables, sugary drinks, sweet and savoury snacks (categories ranged from less than once a week to ≥3 times a day) [12]. Each food category was assigned a score from 1 to 7, with a higher score indicating more frequent consumption of fruits and vegetables, and lower consumption of sugary drinks and snacks. A healthy eating score was derived as a mean of these sub-scores, with a score of 5 or above indicating a healthy diet; 3) Child’s daily physical activity assessed with the question ‘On average, how many minutes of physical activity (described as any activity that increases heart rate and makes the child get out of breath) does your child do?’; children who met the national physical activity recommendation of at least one hour per day [13] were categorised as engaging in adequate physical activity; and 4) Child’s daily screen time (the number of hours spent watching television or playing video games); responses were categorised according to whether children met screen time recommendations of less than two hours per day [14].

In addition to these, the following outcomes were assessed in the parents of overweight and obese children: 1) Parental recognition of their child’s overweight status (child described as ‘overweight’ or ‘very overweight’) in response to the question ‘How would you describe your child’s weight at the moment’; 2) Parental perception of the health risks associated with their child’s overweight status (parent answered ‘yes’ to the question, ‘Do you think your child’s current weight is a health risk?’); 3) Weight-related teasing, assessed using the *Teasing/Marginalisation* subscale from Sizing Them Up, a validated parent-proxy measure of obesity-specific health related quality of life (HRQOL) scale [15]. A score of 50 or higher represented frequent weight-related teasing; and, 4) Child’s self-esteem, assessed using the *Emotional functioning* subscale of the Sizing Them Up scale. A score of 50 or higher indicated frequent episodes of low self-esteem.

At follow-up, all parents were asked whether they had sought further information regarding their child’s weight, from sources including the school nurse, general practitioner (GP), pharmacist, or friends and family. Parents were also asked about the emotions they had experienced in response to the feedback (surprised, guilty, proud, pleased, upset, angry, ashamed, judged, or indifferent). Questionnaire responses were linked to children’s NCMP data, which included anthropometric, ethnicity, and deprivation data (Index of Multiple Deprivation (IMD) score, a measure of local area deprivation based on respondent’s postcode [16]). Anonymised data on weight status, ethnicity, age, and IMD score were obtained for all non-responders for comparison with the study sample.

### Statistical analysis

Analyses were restricted to respondents with longitudinal data (at baseline and at least one follow-up). Where parents completed a questionnaire at both follow-ups, precedence was given to data at one month. The effect of including questionnaire responses at six months was assessed in a sensitivity analysis restricted to responses at one month. We calculated the proportions of parents reporting each outcome at baseline and at follow-up. The difference between pre- and post-feedback proportions was assessed using a McNemar’s test. Analyses of the differences were stratified by socio-demographic characteristics: PCT, child’s sex, school year, ethnicity (white or non-white), deprivation (quintiles of IMD score) and child’s weight category (three categories: healthy weight and underweight combined, overweight, or obese), and by type of feedback among parents of obese children (letter only or letter plus proactive feedback). Differences in the outcomes by socio-demographic characteristics and type of feedback were assessed using chi-squared tests. To account for potential clustering by PCT, multi-variable random effects logistic regression analyses were conducted to assess whether parental recognition of overweight, the associated health risks or lifestyle behaviour changed from baseline to follow-up in the parents of overweight and obese children, with PCT entered into the model. To assess the effect of different types of feedback (letter versus pro-active), interaction terms were included in the regression models to assess potential modification of the main effect by feedback type, gender, ethnicity or deprivation. The effects of seasonality on lifestyle behaviours were explored by comparing differences in outcomes across seasons. All analyses were conducted using Stata 12 (College Station, TX: StataCorp).

## Results

### Characteristics of study sample

Of the 3,397 parents that responded to the baseline questionnaire (response rate: 18.9%), 1,844 (54%) completed a questionnaire at follow-up and were included in the analyses. Compared to all children participating in the NCMP in the five PCTs, the study sample had lower proportions of overweight and obese children, year 6 children, parents from the most deprived areas, and parents of children from ethnic minorities (Table 2). Results of sensitivity analyses which excluded responses at six months follow-up (n = 452, 24.5%) indicated that including responses at six months (where no response at one month was available) did not affect the results, therefore results for the combined sample are presented.

**Table 2:** Baseline characteristics of the sample compared to the total NCMP population

	**Study sample (%) N = 1,844**	**NCMP population (%) N = 18,000**	P*
**Sex**			
Girls	49.7	48.4	0.29
Boys	50.3	51.6	
**Ethnicity**			
White	66.0	54.5	<0.01
Asian	5.5	10.8	
Black	15.7	21.2	
Mixed/other	12.8	13.5	
**School year**			
Reception (4–5 years)	55.5	49.1	<0.01
Year 6 (10–11 years)	44.5	50.9	
**Weight status**			
Underweight	1.9	1.4	<0.01
Healthy weight	82.8	76.5	
Overweight	9.7	12.5	
Obese	5.7	9.6	
**Deprivation quintile**^**†**^			
1 (most deprived)	19.1	20.3	<0.01
2	24.6	28.8	
3	19.9	21.6	
4	16.7	15.7	
5 (least deprived)	19.7	13.7	

*From chi-squared test for differences between groups; ^†^Quintiles based on Index of Multiple Deprivation (IMD).

### Baseline characteristics

The majority of respondents were white (66.0%); 15.7% were Asian, 5.5% were Black, and 12.8% were of mixed or other ethnicity. Of the parents of obese children, 61.9% received proactive feedback in addition to the letter. At baseline, three quarters of parents were able to identify the common health conditions associated with obesity. About half of the children met dietary and screen time recommendations, and a third achieved recommended levels of physical activity. Among parents of overweight and obese children, parental recognition of child overweight was low (14% in overweight, 35% in obese).

### Impact of NCMP feedback

**Figure 1:**
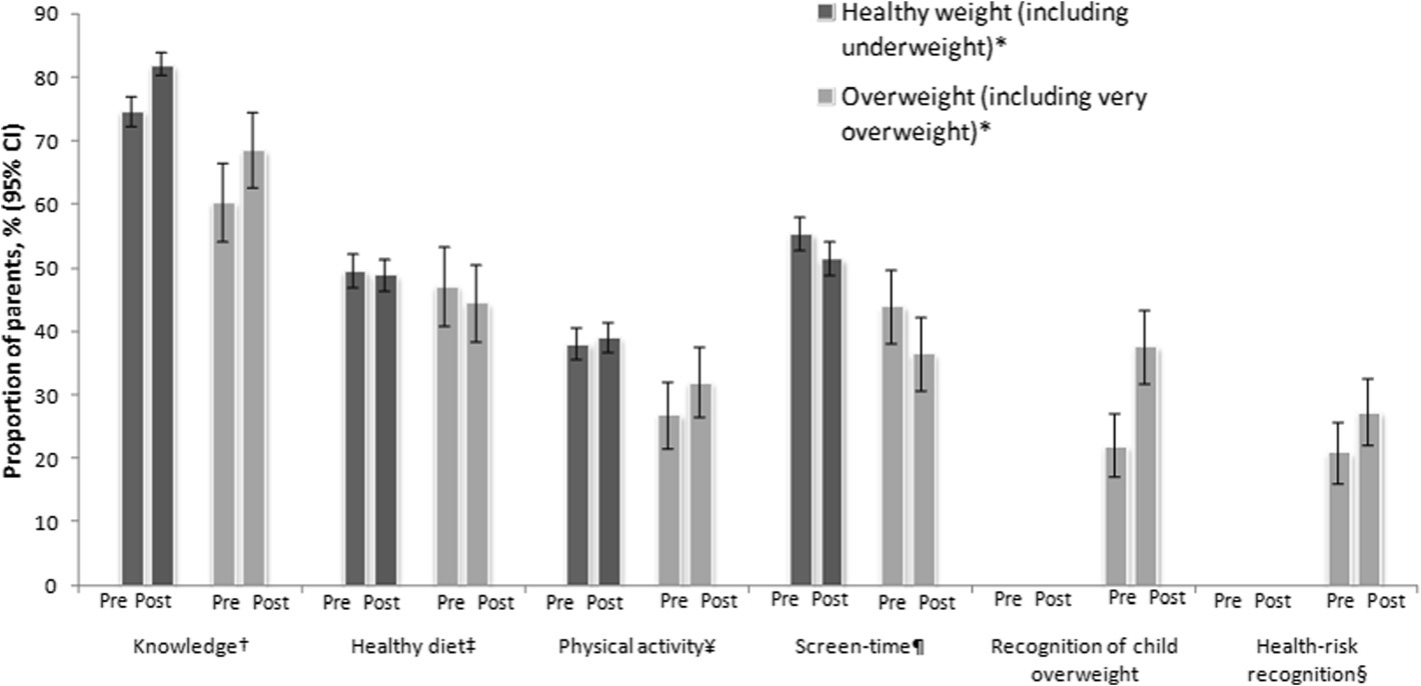
**Parental perceptions and child lifestyle behaviours, (by child’s weight status).** *Healthy weight (including underweight: BMI <85th); Overweight (including obese: BMI ≥ 85th centile); †Knowledge: parental knowledge score ≥4; ‡ Healthy diet: healthy eating score ≥5; ¥ Physical activity: child achieves ≥1 hour physical activity per day; ¶ Screen time: child achieves ≤2 hours screen time per day; § Health-risk recognition: parent of overweight or obese child perceives their child’s weight to pose a health risk.

Parents’ general knowledge about the health risks associated with child overweight increased following weight feedback, with greater increases among parents of overweight and obese children (Table 3, Figure 1). Dietary behaviour was unchanged. The proportion of children meeting physical activity guidelines increased among obese children (difference 12.6%; 95% CI: 2.5 to 22.8), but not among children in the other weight categories. In contrast, the proportion of children engaging in appropriate screen time behaviour was lower after feedback. There was no strong evidence for a seasonal effect on lifestyle behaviours.

**Table 3:** Parental perceptions and child behaviours, before and after weight feedback, by child’s weight status

	**Healthy weight and underweight (n = 1574)**			**Overweight (n = 180)**			**Obese (n = 105)**		
**Outcome**	**Baseline % (95% CI)**	**Follow-up % (95% CI)**	Difference in proportion* % (95% CI)	**Baseline % (95% CI)**	**Follow-up % (95% CI)**	Difference in proportion* % (95% CI)	**Baseline % (95% CI)**	**Follow-up % (95% CI)**	Difference in proportion* % (95% CI)
Good parental knowledge of the health risks of child overweight†	74.8 (72.5 to 77.1)	81.9 (79.9 to 83.4)	7.1 (4.6 to 9.6)	61.9 (54.2 to 69.7)	70.3 (63.1 to 77.6)	8.4 (−0.4 to 17.2)	57.8 (47.4 to 68.2)	65.6 (55.5 to 75.6)	7.8 (−4.5 to 20.1)
Child achieves a healthy diet‡	49.6 (47.0 to 52.2)	48.9 (46.3 to 51.5)	−0.7 (−3.4 to 2.0)	50.6 (42.8 to 58.4)	46.3 (38.5 to 54.1)	−4.3 (−12.7 to 4.0)	41.3 (31.1 to 51.6)	41.3 (31.1 to 51.6)	0 (−10.6 to 10.6)
● Fruit and vegetable consumption (≥5 portions/day)	32.5 (30.2 to 34.8)	33.0 (30.6 to 35.3)	0.5 (−2.0 to 2.8)	27.8(21.2 to 34.4)	25.0 (18.6 to 31.4)	−2.8 (−10.4 to 4.7)	22.9 (14.7 to 31.0)	28.6 (19.8 to 37.4)	5.7 (−3.5 to 14.9)
● Sugar sweetened beverage consumption (<1 per day)	71.0 (68.7 to 73.3)	70.0 (67.7 to 72.4)	−1.0 (−3.5 to 1.5)	77.3 (71.0 to 83.5)	75.6 (69.2 to 82.0)	−1.7 (−9.9 to 6.5)	63.6 (54.0 to 73.3)	66.7 (57.2 to 76.1)	3.0 (−8.6 to 14.7)
Child achieves adequate physical activity (≥1 h per day)	38.1 (35.6 to 40.5)	39.1 (36.7 to 41.5)	1.0 (−1.6 to 3.6)	27.9 (21.1 to 34.7)	28.5 (21.7 to 35.3)	0.6 (−6.1 to 7.3)	25.2 (16.7 to 33.8)	37.9 (28.3 to 47.4)	12.6 (2.5 to 22.8)
Child achieves appropriate screen time behaviour (≤2 h per day)	55.4 (52.9 to 57.9)	51.5 (48.9 to 54.0)	−4.0 (−6.6 to −1.4)	45.5 (38.0 to 52.9)	39.2 (31.9 to 46.5)	−6.3 (−14.2 to 17.3)	41.6 (31.8 to 51.4)	31.7 (22.5 to 40.9)	−9.9 (−20.6 to 0.8)
Parental recognition of child overweight	NA	NA	NA	14.0 (8.8 to 19.3)	25.1 (18.6 to 31.7)	11.1 (4.0 to 18.3)	35.3 (25.9 to 44.7)	58.8 (49.1 to 68.5)	23.5 (12.7 to 34.3)
Parental recognition of the health risks associated with child’s overweight	NA	NA	NA	11.1 (6.4 to 15.9)	18.1 (12.3 to 24.0)	7.0 (1.4 to 12.6)	38.0 (28.3 to 47.7)	43.0 (33.1 to 52.9)	5.0 (−6.9 to 16.9)
Weight-related teasing¥	NA	NA	NA	4.3 (−1.7 to 10.2)	10.6 (1.5 to 19.8)	6.4 (−2.7 to 15.5)	19.0 (0.7 to 37.4)	14.3 (−2.0 to 30.6)	−4.8 (−25.6 to 16.0)
Low self-esteem¶	NA	NA	NA	0	0	0	10.0 (−4.4 to 24.4)	5 (−5.5 to 15.5)	−5.0 (−26.8 to 16.8)

*Difference between baseline and follow-up (proportion at follow-up minus proportion at baseline) from McNemars test for differences; †Knowledge score ≥4; ‡Healthy eating score ≥5. Diet score generated as mean of scores for consumption of fruit and vegetables (higher consumption = higher score) and sugary drinks and sweet and savoury snacks (higher consumption = lower score), range 1–7; ¥Score >50 on teasing/marginalisation subscale of the obesity-specific Health-related Quality of Life (HRQOL) scale; ¶Score >50 on emotional function scale of the obesity-specific HRQOL scale.

FOOTNOTE: Weight feedback varies by child’s weight status and therefore direct comparisons between groups may not be appropriate.

Parents of overweight and obese children were more likely to recognise their child’s weight status following weight feedback, with a greater increase among parents of obese children (23.5% versus 11.1%, p = 0.03). Improvement in parents’ recognition of their own child’s weight-related health risks was modest in both overweight (7.0%) and obese children (5.0%). Despite this, more than a third of parents of overweight and obese children sought further information regarding their child’s weight following feedback. Friends and family (reported by 14.4% of parents) and the internet (9.9%) were the most frequently reported sources of information, followed by the GP (8.9%) and school nurse (8.4%). Weight-related teasing and low self-esteem were more prevalent in obese children compared to overweight children at both time points; however, there were no apparent effects of feedback. The proportion of children experiencing weight-related teasing and low self-esteem did not change substantially between baseline and follow-up.

### Effects of socio-demographic characteristics and type of feedback

Among the parents of children from ethnic minority groups, there was a larger increase in the proportion that recognised the health risks associated with their child’s overweight status (compared to white parents, p < 0.01) Table 4. After accounting for clustering by PCT, the parents of children from ethnic minority groups were 8 times more likely to have changed their recognition of the health risks associated with child overweight compared to the parents of white children (OR: 8.6, 95% CI: 1.9, 39.8). There were no apparent socio-demographic effects on lifestyle behaviours.

**Table 4:** Change in parental perceptions and obesity-related behaviours following weight feedback, among parents of overweight and obese children, (by socio-demographic characteristics and the type of feedback received)

**Characteristic**	**Parental recognition of overweight**		**Parental recognition of the health risks associated with child’s overweight**		**Child achieves a healthy diet**		**Child performs adequate physical activity**	
	Difference in proportion % (95% CI)*	**P value**^**†**^	Difference in proportion % (95% CI)*	**P value**^**†**^	Difference in proportion % (95% CI)*	**P value**^**†**^	Difference in proportion (95% CI)*	**P value**^**†**^
**Ethnicity**								
White	12.6 (5.6 to 19.6)	0.24	−3.3 (−8.7 to 2.1)	<0.01	−2.9 (−10.9 to 5.1)	0.98	7.3 (0.3 to 14.4)	0.37
Non-white	19.3 (10.1 to 28.6)		17.9 (8.8 to 27.1)		−2.7 (−12.0 to 6.6)		2.5 (−5.7 to 10.6)	
**Sex**								0.27
Girls	14.8 (6.4 to 23.3)	0.75	5.8 (−2.3 to 14.1)	0.88	−3.9 (−12.2 to 4.4)	0.71	2.2 (−3.7 to 8.1)	
Boys	16.7 (9.2 to 24.2)		6.7 (0.4 to 13.0)		−1.6 (−10.5 to 7.3)		8.1 (−.07 to 16.7)	
**School year**								0.76
Reception (4–5 yrs)	16.9 (9.3 to 24.5)	0.69	8.1 (1.2 to 15.1)	0.48	−10.5 (−18.9 to −2.1)	0.01	5.9 (−1.5 to 13.4)	
Year 6 (10–11 yrs)	14.6 (6.3 to 22.9)		4.4 (−3.3 to 12.1)		4.6 (−4.0 to 13.2)		4.3 (−3.2 to 11.8)	
**Deprivation quintile**^**‡**^								
1 (most deprived)	13.3 (0.3 to 26.3)	0.54	3.8 (−5.2 to 12.7)	0.41	6.8 (−4.4 to 18.1)	0.82	6.3 (−3.3 to 15.8)	0.58
2	14.8 (4.3 to 25.4)		5.6 (−3.0 to 14.1)		−5.9 (−16.1 to 4.3)		10.9 (0.03 to 21.7)	
3	26.4 (13.0 to 29.8)		13.0 (−0.07 to 26.0)		−3.8 (−17.3 to 9.6)		−1.8 (−11.5 to 7.9)	
4	13.6 (0.4 to 27.7)		11.4 (−0.4 to 23.1)		−4.8 (−22.9 to 13.4)		6.8 (−6.9 to 20.6)	
5	11.1 (5.6 to 27.8)		−3.9 (−17.7 to 10.1)		−4.0 (−22.8 to 14.8)		11.1 (−8.9 to 31.1)	
**Feedback type**^**¥**^								0.69
Letter	10.0 (−7.4 to 27.4)	0.03	−7.9 (−27.2 to 11.4)	0.07	−8.8 (−24.6 to 6.9)	0.17	15.0 (−2.1 to 32.1)	
Proactive	32.3 (20.2 to 44.2)		12.9 (−0.5 to 26.3)		5.2 (−7.3 to 17.7)		11.1 (−0.07 to 22.2)	

*Difference between baseline and follow-up (proportion at follow-up minus proportion at baseline); ^†^P-value from Chi-squared test for differences; ‡Quintiles based on index of multiple deprivation (IMD); ^¥^Analyses restricted to obese children (N = 105).

There was some evidence to suggest that proactive feedback may be more effective than a letter alone, with greater improvements in parental recognition of child overweight (32% versus 10%, p < 0.01) and the associated health risks (13% versus −8%, p = 0.07) among the parents of obese children living in PCT areas with a proactive feedback program. However, in regression analyses, there was no evidence that in the parents of obese children, pro-active feedback had a greater impact on a change in recognition of overweight (adjusted OR: 0.13, 95% CI: 0.02, 6.9), the associated health risks (adjusted OR: 0.35, 95% CI: 0.02, 6.3), diet (adjusted OR: 3.6, 95% CI: 0.32, 42.6) or physical activity (adjusted OR: 0.68, 95% CI: 0.05, 9.1). Parents expressed a strong preference for written feedback, with 84.4% preferring feedback by letter compared to 3.0% by telephone. There were no differences in outcomes by child’s sex or deprivation.

### Parental experience

Following receipt of NCMP feedback, more than one fifth of parents of non-healthy weight children reported feeling upset (22.7% of underweight, 21% of overweight, and 24.1% of obese children), compared to 0.5% of parents with healthy weight children. Less than 1% of parents of healthy weight and underweight children reported guilt or anger, while 15.4% of parents of overweight and obese children reported feeling guilt and 14.8% anger. Despite this, 87.2% of all parents found the feedback to be helpful. The majority of parents (70.0%) reported that they would encourage future participation in the NCMP for their child or child’s siblings. Only 1.8% of parents stated that they would withdraw their child from the programme in the future.

## Discussion

We report findings from the first large-scale prospective evaluation of a national childhood weight screening and feedback programme. Parents’ general knowledge about the health issues associated with child overweight increased following weight feedback, as did the proportion of parents that recognised their child’s overweight status. More than a third of parents of overweight children reported seeking further information regarding their child’s weight, but this did not translate into notable changes in lifestyle behaviours. There was no evidence of an effect on child weight-related teasing and self-esteem, and the majority of parents found weight feedback to be helpful. There was some evidence to suggest that weight feedback was more effective for non-white parents and when supplemented by proactive telephone calls, although parents preferred written feedback.

This study was conducted in a large, population-based and socio-demographically diverse sample, which enabled examination of the effects of weight feedback in different weight and socio-demographic groups. A short time interval was selected between baseline and follow-up questionnaires (~3 months) to minimise potential changes in outcomes that may have occurred independently of the intervention. The main limitation of this study was the low response rate and high attrition, which raise the possibility of a biased sample; it is plausible that parents who are more engaged with issues relating to their child’s health would be more likely to participate in the study and respond to feedback, than parents who are not engaged with these issues. Comparison of the study sample with all children taking part in the NCMP in the five PCTs revealed a slight underrepresentation of children from ethnic minorities, children from year 6, children from deprived areas and overweight/obese children in our sample, which may limit the generalisability of the study findings to the NCMP population in the five PCTs. However, the lifestyle behaviours of the study sample were similar to those previously observed in national surveys of primary school-aged children [17-19]. Furthermore, we found no clear effects of socio-demographics on outcome variables, except for ethnicity. This would suggest that the only consequence of any limitations of the representativeness of our study sample may be a small underestimation of the weight feedback effect. In order to keep the questionnaires concise, brief measures of behaviour and potential harms were used, not all of which are validated. Self-reported measures of lifestyle behaviours may be subject to misreporting, in particular social desirability bias. However, a lack of improvement in reported lifestyle behaviours post-feedback argues against this being an issue [20].

The dietary measures used have been previously assessed using test-retest methods and found to be reasonably reliable [12]. In addition, parental perceptions of overweight and health risk were evaluated using questions that have been used in previous evaluations of weight feedback [5].

As demonstrated in other studies, we found that parents had good general awareness of the health risks associated with childhood overweight [21,22], which further increased following feedback. We also found that the proportion of parents of overweight and obese children that recognised their child’s weight status increased following receipt of weight feedback. However, despite the observed increase in recognition and following receipt of detailed weight feedback, less than half of parents of overweight and obese children perceived their child to be overweight [3]. A qualitative study of parents receiving NCMP feedback found that parents considered many different factors other than weight, when determining if their child was overweight in response to weight feedback [23]. These factors included parents disagreeing with the feedback, attributing the child’s weight to puppy fat or genetics, and being more concerned with the child’s health and happiness than their weight. These factors should be considered when developing weight feedback.

The proportion of parents of overweight and obese children that perceived their child’s weight status to be a health risk was low at follow-up, but higher than at baseline; those parents who did change their perception of their child’s health may be more likely to engage with child weight issues [4,24]. The parents of ethnic minority children demonstrated a larger change in recognition of the health risks than parents of white children. A similar effect has been observed in other studies, with ethnic minority groups reporting greater changes to lifestyle and plans for help-seeking following weight feedback [25,26]. Although this variation by ethnic group requires further investigation, these findings are encouraging, as many ethnic minority groups are at increased risk of obesity and its co-morbidities [27], and typically have lower rates of participation in obesity prevention initiatives compared to white populations [28,29].

In this study, there was no observed effect of weight feedback on dietary behaviours, while a positive effect on physical activity was only observed among obese children. A previous evaluation of NCMP feedback showed that many parents plan to make changes to behaviour following feedback [8], but our study indicates that actual behaviour changes may be small and limited to certain groups. Given the low-intensity nature of routine weight feedback as an intervention, it may be unrealistic to expect large changes in behaviour [30]. In the case of screen time behaviour, fewer children met the screen time recommendations after feedback than before. A possible explanation for this unexpected finding is that the information provided with weight feedback does not make explicit recommendations about screen time.

More than a third of parents of overweight and obese children sought further information regarding their child’s weight in response to the feedback. Informal sources of help (friends and family, the internet) were most commonly consulted, suggesting that parents may feel more comfortable approaching these than more official sources of information. An implication of this finding is that the quality of information may be difficult to monitor.

More than half of parents reported discussing the feedback with their child; however, we found no adverse effects on self-esteem and weight-related teasing in overweight and obese children [5]. The majority of parents reported that they found the feedback to be helpful and would encourage future participation in the NCMP. A small number of parents reported feeling upset or angry in response to the feedback. Consideration of the sensitive nature of weight issues should be a priority when devising feedback [31].

There was some evidence to suggest that weight feedback supplemented by telephone calls may be more effective in increasing parental recognition of child overweight and the associated health risks than written feedback alone. Analysis of the effects of proactive feedback was limited by the small number of parents that received this intervention. In general, more intensive behaviour change interventions have greater success and this may be applicable in the context of weight feedback provision [30,32]. However, the potential benefits of proactive feedback must be balanced against the additional costs, parents’ preference for written feedback, and difficulties in contacting parents by telephone.

Parents appear to have good general knowledge of the health risks associated with childhood overweight, but many do not associate these risks with their own overweight child. Research to understand how parental perceptions of their child’s weight status and health are formed may identify ways to more effectively communicate information to parents. Further work is required to identify how parents can be encouraged and supported in making and maintaining lifestyle changes, and also to explore the type of health information that parents access from informal sources. The capacity of local services and the expertise of staff to cope with increased demand that may result from weight screening and feedback programmes also need to be assessed.

## Conclusions

Weight feedback as part of the Department of Health for England’s National Child Measurement Programme appears to benefit parental awareness and recognition of childhood overweight in some parents with minimal harms, limited to a proportion of parents experiencing upset or anger. The NCMP is likely to incur substantial delivery costs and the mixed results of this study suggest that although weight feedback may have a role as part of a multi-faceted strategy to ease the burden of child obesity, other initiatives may be required for lifestyle behaviour change.

## Competing interests

AK is also Director of Public Health Strategy, Medical Director and R&D Director at Public Health England. The views expressed in this paper are those of the authors, and are not intended to represent the views of Public Health England. The other authors have no competing interest relevant to this article to disclose.

## Authors’ contributions

SK and RV were responsible for the study conception and design, and provided critical manuscript revision and approval. CF managed the study, coordinated the data collection, undertook the statistical analyses, and drafted and revised the manuscript. MHP participated in the statistical analysis, and drafting and revision of the manuscript. JB, AS, AK, HC, SK and SM participated in developing the study and data collection instruments, and critically reviewed the manuscript. All authors read and approved the final manuscript.

## Pre-publication history

The pre-publication history for this paper can be accessed here:

http://www.biomedcentral.com/1471-2458/14/549/prepub
